# Methods for Obtaining and Determination of Squalene from Natural Sources

**DOI:** 10.1155/2015/367202

**Published:** 2015-01-28

**Authors:** Ovidiu Popa, Narcisa Elena Băbeanu, Ioana Popa, Sultana Niță, Cristina Elena Dinu-Pârvu

**Affiliations:** ^1^Department of Industrial Biotechnologies, Faculty of Biotechnologies, University of Agricultural Sciences and Veterinary Medicine Bucharest, 59 Marasti, District 1, 011464 Bucharest, Romania; ^2^Department of Physical-Chemical Analysis and Quality Control, National Institute for Chemical Pharmaceutical Research and Development, 112 Vitan, District 3, 031299 Bucharest, Romania; ^3^Physical and Colloidal Chemistry, Department 1 (Fundamental Sciences), Faculty of Pharmacy, Carol Davila University of Medicine and Pharmacy, 6 Traian Vuia Street, District 2, 020956 Bucharest, Romania

## Abstract

Squalene is a natural dehydrotriterpenic hydrocarbon (C_30_H_50_) with six double bonds, known as an intermediate in the biosynthesis of phytosterol or cholesterol in plants or animals. We have briefly reviewed the natural sources for squalene and focused on the main methods and techniques to obtain and to determine it. Some of its applications in different fields of human activity are also mentioned.

## 1. Introduction

Squalene is a triterpene with the formula C_30_H_50_, an intermediate for the biosynthesis of phytosterol or cholesterol in plants/animals and humans, widespread in animal and vegetal kingdom. Scientists have discovered that, at the moment life appeared on Earth, microorganisms, and later in Precambrian the cells' membrane of higher organisms, contained, in a great proportion, squalene, a substance likely to be essential to their survival in that hostile environment free of oxygen.

Until the last decades of the *XX*th century, it was not known that squalene exists in small amounts also in the human body. Among the human being, it seems that the newborn have the greatest concentration of squalene in their blood, but the reserve begins to drop suddenly between 30 and 40 years. In human body squalene is synthesized by the liver and is secreted in large quantities by the sebaceous glands. It is transported in the blood by the small and very small density lipoproteins [1]. It is interesting to notice that squalene represents 12% of the lipids secreted by the sebaceous glands and it is not transformed in cholesterol [2, 3]. The highest squalene concentrations in human is met in skin lipids, about 500 *μ*g/g, and in the adipose tissue, 300 *μ*g/g [4]; while in organs where the active biosynthesis takes place, the concentration is much smaller, as in liver 75 *μ*g/g or small intestine 42 *μ*g/g [5].

Squalene was discovered in 1906 by the Japanese researcher Dr. Mitsumaru Tsujimoto, an expert in oils and fats at Tokyo Industrial Testing Station. He separated the unsaponifiable fraction from the shark liver oil “*kuroko-zame*” and discovered the existence of a highly unsaturated hydrocarbon [6]. Ten years later, Tsujimoto succeeded to obtain by fractional vacuum of the liver oil from two deep-sea shark species an unsaturated hydrocarbon, with the chemical formula C_30_H_50_, which he named “squalene” [7]. The name came from the denomination of the sharks' family: Squalidae.

**Scheme 1:**
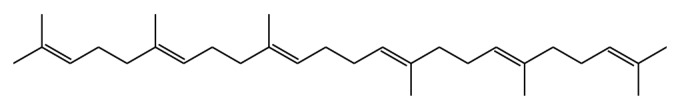
(*E*)-2,6,10,15,19,23-Hexamethyl-2,6,10,14,18,22-tetracosahexaene.

Subsequent research papers [8–11] have confirmed the mentioned chemical formula proposed by Tsujimoto, squalene having the chemical structure shown in Scheme 1 and the chemical and physical properties presented in Table 1 [12].

The greatest concentration of squalene in the living world is met in the liver of certain species of fish, especially sharks living in sea at depth under 400 m. Tsujimoto's studies [13] showed that squalene was present in 16 from the 36 studied shark species from Japanese sea waters.

In the case of deep-sea sharks, the liver is the main organ for lipids' storage, being in the same time an energy source and means for adjusting the buoyancy. In their case, the unsaponifiable matter represents 50–80% of the liver, the great majority thereof being squalene. For example,* Centrophorus artomarginatus* deep-sea sharks which live in waters at 600 to 1000 m depth, without sunlight, manage to survive where pressure is consistently high and the oxygen supply is very poor, due to this compound from their liver, which accounts for 25% to 30% of their total body weight.

The liver of the shark* Centrophorus squamosus* represents 18.1% from the body mass; 77.2% by weight of the liver composition is oil and the squalene concentration in this oil is 79.6%. Similar values have been obtained for* Centroscymnus crepidater*. An interesting discovery has been made—squalene can be found in higher amounts not only in the liver of some shark species but also in other organs thereof. As proved by a Norwegian study [14], squalene is present in great concentrations also in other organs, except the liver, the highest squalene content for the species* Centrophorus squamosus* (Centrophoridae) being 70.60 ± 0.81% in the liver, but also 52.45 ± 0.64% in the stomach, 45.64 ± 10.59% in the pancreas, 42.98 ± 7.30% in the heart, 30.06 ± 16.10% in the spleen, and 5.30 ± 4.55% in the kidneys. The study has been conducted on the mentioned shark species, which live at 800 to 1,500 meters depth, in North Atlantic ocean. Variability of data for the individuals from the same species has been proved to be high, which was due to different age, sex, geographical parameters, and season variation.

Shark liver oil remains the richest natural source of squalene. A limitative reason in the use of this natural source for squalene is represented by the presence of different persistent organic pollutants (POPs) in the sea environment, like PCB (polychlorinated biphenyl), PBDE (polybrominated diphenylether), organochlorine pesticides, polycyclic aromatic hydrocarbons, dioxin, and heavy metals, together with the concern in preservation of marine life.

However, more recent studies [15] show that the POP level in Atlantic grown salmon is under the limit established by the World Health Organization and the European Authority for Food Safety.

Despite the questionable presence of POPs in squalene obtained from shark liver oil, its use in pharmaceutical industry can raise problems due to the possible presence of various pathogens with which sharks could be infected and so the infection is transmitted to humans.

Anyway, the intensive fishing of these sharks puts in danger the existence of these species, many of them being close to extinction as their reproductive cycle is quite long and the growth is slow. That is why Europe drastically reduced in the last period the fishing quotas for these shark species and at the same time the European market seems to change to squalene of vegetal origin, at least in the cosmetics.

For all these reasons there is today a great interest in finding new natural sources for squalene, especially vegetal ones.

Squalene was also identified in many plant oils in different concentrations. Chronologically, the first vegetable oil in which it was found was the olive oil [16]. More recently, Frega et al. [17] determined the squalene concentration in olive oil to be 564 mg/100 g, in soybean oil 9.9 mg/100 g, in grape seed oil 14.1 mg/100 g, in hazelnuts oil 27.9 mg/100 g, in peanuts oil 27.4 mg/100 g, and in corn oil 27.4 mg/100 g. The concentration range for squalene in vegetable oils produced on a large scale in Europe is 0–0.19 g/kg in sunflower oil, 0.03–0.2 g/kg in soybean oil, 0.1–0.17 g/kg in corn oil [18], and 1.7–4.6 g/kg in olive oil [19]. A pseudograin* Amaranthus* sp., more recently introduced in Europe, is now known to be the plant with mostly the highest concentration of squalene in vegetal world, 4.16 g squalene/kg seed [20]. An extensive study conducted on 104 genotypes of 30 species of* Amaranthus* revealed concentrations of squalene between 10.4 and 73.0 g/kg amaranth oil [21].

## 2. Separation of Squalene from Vegetable Sources

**Figure 1:**
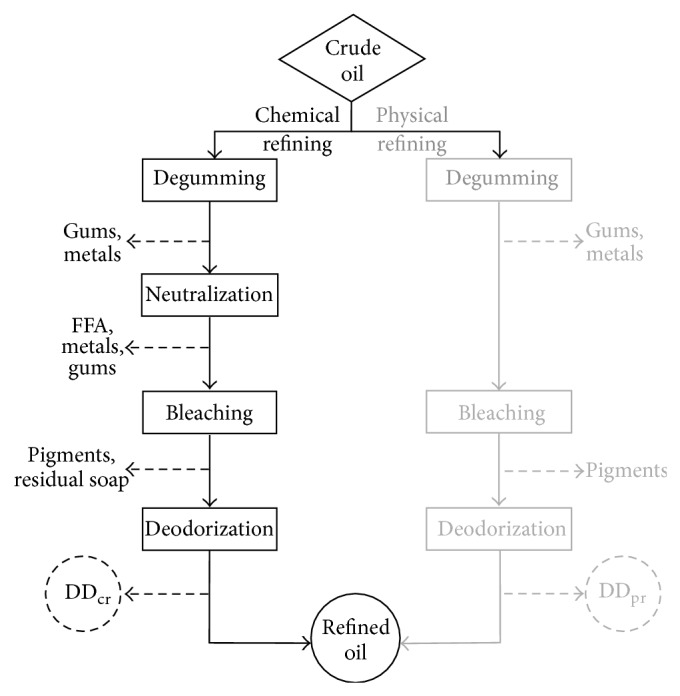
General flow sheet in the process of refining crude vegetal oils.

Most plant oils are obtained by mechanical pressing or by chemical extraction with organic solvents, like hexane. This way, there are two valuable components obtained: the oil and flour with a high protein content. While the obtaining of plant oils by mechanical pressing leads to a maximum yield of 80%, by chemical extraction (using solvents) the yield could be even more than 98%. Furthermore the concentration of the residual oil in the cake resulting from pressing may be 2-3%, and, in the case of the chemical extraction, it is less than 0.5%. The oil refining is necessary in almost all the cases, except the extra virgin oils. Refining aims at the removal of undesired substances with unfavourable effects on the oils' taste, smell, aspect, or stability (phospholipids, diacylglycerols, free fatty acids, pigments, and oxidation products). By refining, the oils' stability and their organoleptic characteristics are improved, but during the various physicochemical treatments, valuable minor components are also removed (antioxidants and vitamins). The influence of different refining processes on the concentration of squalene in olive oil was reported [22]: the decrease due to physical refining was 13.0%, discolouring 7.0%, and deodorization 15.6%. Due to the small concentration of squalene in vegetable oils, this relatively low decrease could become relevant for the final productivity of the process. Independent of the vegetal edible oil, this will be subjected to a multiple step process for removing the undesired substances in an edible oil, presented in Figure 1.

Following this refining process a deodorizer distillate will result as by-product, source of many valuable compounds (tocopherols, squalene).

The chemical composition of the olive oil reveals a saponifiable fraction which represents 98-99% of the total weight and an unsaponifiable fraction which accounts for 0.5–2% of the total weight. This last one contains several active components, as squalene, *β*-carotene, tocopherols, and tocotrienols. By nowadays technology, it is not economic to separate these valuable constituents directly from the vegetal oils, as the concentration is too small. But, the by-products obtained in the process of oils' refining, like the deodorizer distillate, contain 15 to 30% unsaponifiable fraction, with a concentration of up to 80% squalene, depending on several factors, representing an economically valid source for squalene.

Several research studies [23, 24] on the squalene concentration in the olive oil have showed that this hydrocarbon represents over 50% of the unsaponifiable fraction and up to 90% of the total hydrocarbons content thereof. Anyway, the final concentration is significantly influenced by the methods of extraction [25] and oil refining [26]. Commercially available methods for squalene extraction from olive oil yield to a vegetable squalene with around 97.5% purity, while squalene obtained from shark liver oil can reach 99.9% purity.

Palm oil contains 250–540 mg/L squalene [27], larger concentrations (2,400–13,500 mg/L) being found in palm oil deodorizer distillate (PODD). If the refining is made by physical methods, the distillate resulting from deacidification and deodorization contains about 1% squalene [28].

The greatest concentration of squalene in vegetal kingdom is met in the oil extracted from* Amaranthus* sp.* Amaranthus* is a pseudograin having seeds and leafs with a great content of oil for a cereal, but significantly lower than oilseed plants, like soybean. The lipid is mainly contained in the coat-embryo fraction of the seed, representing about 25% of its total weight. Even the oil content is smaller than that in specific oilseed plants, which is very rich in squalene, its concentration in amaranth oil being several times bigger than that in olive oil. Result of a research project about* Amaranthus* as an alternative crop in Central Europe, published data, confirms an average concentration of squalene in amaranth oil of about 7-8% (wt/wt) comparative with an average of 1% in olive oil [29]. The above-mentioned vegetable oils are an important source of minor valuable compounds, as it is shown in Table 2.

The concentration of all these minor constituents is in the most cases too small that the direct separation thereof from the oil is economically effective. That is why the deodorization distillates obtained as a by-product in the process of oil refining are a valid solution.

Olive oil deodorization distillate (OODD) is an important natural source for squalene, its content being bigger than that in other distillates obtained by refining vegetable oils [34]. Akgün [35] reported that he worked with an OODD composed of 41.55% unsaponifiables, which are represented by 58.02 of squalene and succeeded by supercritical fluid extraction with CO_2_ to concentrate squalene from an initial amount of 24.10% to 66.57% (wt/wt). Besides squalene, OODD contains other precious components, like fatty acids, sterols, and tocopherols. The need for squalene from vegetal sources is not satisfied only by the olive oil and its by-products from the distillation, so extensive researches are being conducted all over the world to identify new valuable vegetal sources and new methods to obtain squalene, less complicated and cheaper.

Another valuable vegetal source of squalene is the soybean oil deodorizer distillate (SODD), a by-product obtained in the deodorization step of soybean oil refining. Being an important oilseed crop, soybean contains 18–20% oil and 40% protein.

Depending on the soybean origin, SODD could be a precious source of active substances, as tocopherols, phytosterols, free fatty acids, or squalene [36–38]; the total concentration of valuable products could reach up to 32% (wt/wt). General chemical composition for deodorizer distillates obtained in the process of oils' refining is presented in Table 3.

Most of the methods for the fractionation of the oil containing squalene presented in the state-of-the-art literature were designed for analytical purposes.

US Patent no. 7161055B2 [41] describes a process for the separation and recovery of minor components from vegetable oils, especially palm oil. Esterified palm oil is subject to molecular distillation yielding to a concentrate rich in minor components, like tocopherols and squalene.

The direct distillation of vegetable oils is not a suitable method to obtain squalene, as it is thermolabile due to its unsaturated linear chain. The literature in the field mentions some other methods for the isolation and/or fractionation of lipid fraction and purification of squalene, which can be applied also in industry. They could be divided into two main types:

- organic solvent extraction, like extraction with hexane, followed by degumming and deacidification, if it is desired, and finally, the mixture is subject to molecular distillation;
- oil extraction from seeds and fractionation thereof with a supercritical fluid, in most cases this is CO_2_, so called SFE—supercritical fluid extraction, a method preferred in the last years but still expensive at industrial level.

After the oil extraction, the separation of miscella could be done also by specific composite membranes [42].

One of the first experimental reports on the squalene manufacture from Amaranth seeds was published in 1987 [43]. It mentions the extraction with hexane, followed by the solvent removal, degumming, and waxes removal. Squalene was finally separated and purified by vacuum distillation. If the oil subject to fractionation is not previously deacidified, the fatty acids leave together with squalene from distillation, leading to the lowering of the squalene concentration in distillate and to the obtaining of a semisolid product at room temperature. Carrying out the distillation at 180°C and 3 mtorr, a sevenfold concentration of squalene was obtained [44], with a recovery yield of 76%. When an alkali-refined amaranth oil was used, a ninefold increase of squalene concentration was reported, but the recovery yield was slightly lower, 67.8%.

In order to avoid the use of toxic solvents like hexane and the oil exposure to high temperatures for long periods of time, today is preferred a “green extraction technology”, Supercritical Fluid Extraction (SFE) with or without cosolvent. As solvent, supercritical carbon dioxide (SC-CO_2_) is usually used because of its inertness, nontoxicity, high volatility, and low cost. Due to the relatively low critical temperature of CO_2_, SFE-CO_2_ is suitable for the extraction of thermally labile substances. Advantages of this extraction method can be mentioned: the high purity of the end product and the short processing time due to the fact that extraction and concentration are carried out in one step. This process yields to high quality squalene without use of toxic unallowable solvents. The efficiency of this extraction method can be improved, for example, if 10–15% ethanol is used as a cosolvent to increase the yield and to extract also polar substances. Anyway it is not economically effective to use SFE for direct extraction of squalene from seeds; it is suitable only for the extraction of squalene from the plant oil.

The case of oil extraction from amaranth seed is more particular as the seed has very small dimensions and also seed's shell is not thick, so the seed crushing is sufficient for the oil extraction and consequently the milling time has no great importance on the final yield [45]. Other authors [46, 47] preferred to fractionate, by means of sieves or pneumatically, the whole seeds into one fraction enriched in protein and oil and another one, with a great concentration of starch. The final efficiency of the process depends eventually on the economic factors.

As a disadvantage of SFE-CO_2_ method the expensive cost of the equipment and the complexity of the operating parameters (high-pressure operation) can be mentioned.

Catchpole et al. [48] used for lipid extraction with supercritical CO_2_, from shark liver oil and OODD, at laboratory and pilot scale, packed columns and static mixers. In the case of shark liver oil, the separation factor for squalene was high, with the mention that, at laboratory level, the separation degree was superior for the packed column to that obtained for static mixer. The separation factor for OODD was lower than the one obtained for shark liver oil and an additional disadvantage was the need for supplementary steps of purification in order to obtain similar purity of the final product.

Czaplicki et al. [49] studied the content of squalene from amaranth oil obtained by three different methods, supercritical fluid extraction, cold pressing extraction, and extraction with chloroform/methanol. The best results expressed as squalene concentration were obtained by SFE, as it is shown in Table 4.

Another comparative data [45] for squalene extraction from amaranth oil by SC-CO_2_ extraction (50°C, 200 bar, 2.0 SL/min–2 h) and Soxhlet extraction with petroleum ether show the highest concentration of squalene in extract, that is, 15.3% (wt), for the first method, versus 6.0% (wt) in the classical method. Anyway, the extraction yield was almost the same in both procedures, around 0.3 g squalene/100 g grain, but the yield for oil extraction was greater for Soxhlet extraction, 4.98 g oil/100 g seeds, comparative with 2.07 g oil/100 g seeds for SCFE. Other authors [50] present other values for squalene extraction with hexane (Soxhlet), 0.238 g squalene/100 g seeds versus SC-CO_2_ extraction (110 min), 0.307 g squalene/100 g seeds, proving also in this case a higher concentration for the SC-CO_2_ extraction, although the extraction yield for oil has only a slight greater value for the classical Soxhlet extraction, 4.70 versus 4.11 g oil/100 g seeds, for SCFE. The differences between the various data in the literature could be explained by the specific characteristics of each plant variety.

A synthetic overview of the methods presented in the literature for squalene obtaining from natural sources is presented in Table 5.

## 3. Quantitative Determination of Squalene

The modern analytic methods used for the quantitative determination of squalene are the chromatographic ones: gas chromatography (GC) alone or coupled with mass spectrometry (GC/MS) or high performance liquid chromatography (HPLC) alone or coupled with mass spectrometry (HPLC/MS).

Bueno determined squalene in the hydrocarbon fraction extracted from olive oil of Extremadura region in Spain [66], using a GC-FID, with TRB-5 stationery phase and a temperature regime in two stages; by this method there were quantified alkanes, alkenes, and sesquiterpenes from olive oil samples.

As an example for HPLC methods HPLC-RID (refractive index detector) can be mentioned with reverse phase (RP) [67], the mobile phase acetone-acetonitrile (1 : 1) which permits the squalene determination in the presence of triglycerides.

The deodorizer distillate, by-product obtained during vegetal oil distillation, is a cheap and, at the same time, rich source of valuable compounds, including squalene. The analysis of the deodorizer distillate is a challenging problem. The quantification of the compounds present in DD by direct analysis is often difficult, especially when the constituents of DD overlap are present in very low concentrations. This requires a pretreatment step to eliminate the interfering substances. Usually, pretreatment involves the saponification of the distillate and the extraction of the unsaponifiable matters, followed by a chromatographic separation by column with different fillers, depending on the composition of DD and the desired compounds to be separated. Quantification can be made by means of GC coupled with HPLC or MS. Saponification prior to GC analysis could cause degradation of bioactive compounds from DD, such as squalene, tocopherols, and FASEs, a problem which has to be taken into account.

Consequently methods using a preliminary fractionation of samples, procedure which simplifies the analysis and shortens its duration, have been developed. The fractional crystallization is a mild process often employed in industry for the modification of fats. Nenadis and Tsimidou [68] reported a preliminary fractional crystallization of samples as a rapid, simple, and low-cost method for determination of squalene from virgin olive oil. Before chromatography, they subjected the samples to a fractional crystallization, following several steps: the oil is dissolved and shaken in a mixture methanol-acetone (7 : 3, v/v) and then kept for several hours at −22°C. After filtration and removal of the solvent at low pressure, the sample is dissolved in the mobile phase acetone-acetonitrile (4 : 6, v/v) and chromatographically separated by RP-HPLC with RID, with the detection of squalene at 208 nm. The method permits the simultaneously determination of squalene and TGA, which is most desired in olive oil industry and the duration is three times shorter than that in classic chromatographic methods.

Quantitative determination of squalene from plant oils can be performed also on a chromatographic column LiChrosper 100 (250 mm × 4 mm × 5 *μ*m), mobile phase acetone-acetonitrile (70 : 30, v/v), and RID [69]. In order to determine squalene MS can be also used if the sample contains a great quantity of triglycerides.

Another method based on exclusion chromatography with RI detector [70] is proposed for the simultaneous determination of mono-, di-, triglycerides and squalene; the samples in tetrahydrofurane are analysed on three Ultrastyragel columns of 50, 100, and 500 Å (25 cm × 0.77 cm), filled with styren-divinylbenzene copolymer and connected in series.

Several authors reported the use of UV and GC-DAD (diode array detector) for squalene determination. Sun et al. [44] proposed a combined method for separation and determination by HPLC/UV (*λ*: 214 nm—squalene; 280 nm—tocopherol) for the simultaneous quantification of squalene and tocopherol from amaranth oil. The mobile phase used for the squalene identification was methanol/i-propanol/acetic acid (91.95/8/0.05, v/v/v), and flow rate was 1.2 mL/min.

In an extensive study [71], squalene and its oxidation products have been identified and analysed by LC/APCI-MS (Liquid Chromatography/Atmospheric Pressure Chemical Ionization-Mass Spectrometry). The method allows the determination of squalene and squalene epoxide and its hydroperoxides in latent fingerprints. The operating conditions were liquid chromatograph coupled with ion trap mass spectrometer; Zorbax Eclipse XDB-C8 column (150 mm × 4.6 mm, 5 *μ*m), sample dissolved in acetonitrile; elution with gradient acetonitrile : water (1 : 1, v/v) for 10 min, linear gradient of up to acetonitrile 100% in 15 min, acetonitrile for 15 min; MS parameters: vaporization temperature 500°C, electric discharge 4.00 *μ*A, capillary temperature 200°C, and capillary voltage 12 V, FULL SCAN detection 200–800 amu, SIM at 409, 425, and 443 amu.

In Table 6 there are several analytical methods reviewed for the determination of squalene in the presence of other active substances present in natural oils.

## 4. Applications

Squalene comes into attention of the scientific world due to the beneficial effects of some natural products containing it, observed on the human health.

It is known that squalene is the main component of the shark liver oil. From ancient times, fishermen all over the world benefited from the wonderful properties of the oil extracted from the liver of sharks living beneath 1,000 m. The shoguns from ancient Japan recognized the benefits of the deep-sea sharks liver oil, as a source of power, force, energy, and vitality, calling it “Tokubetsu no Miyage,” meaning “precious gift.” This oil was also known and used by coastal residents and fishermen in Micronesia, who referred to it as “miraculous oil.” Locals from Japanese peninsula Izu called this shark liver oil “Samedawa,” meaning “cure-all.” They accustomed themselves to use it to cure a wide range of conditions [82].

On the other hand, the other major traditional natural source for squalene, the olive oil, brought into attention of the scientific community the healthy properties of an olive oil based diet. The epidemiological evidence of a lower incidence of CHD (cardiovascular heart disease) and certain cancers in the Mediterranean area [83] stimulated the researches on the potentially protective action of the olive oil's minor constituents.

Comparative studies were made regarding the incidence of certain cancers between Mediterranean countries like Greece, Spain, or Italy, where the olive oil is a constant part of the daily diet, and Scandinavian countries and USA. While in Mediterranean area the daily uptake of squalene (from olive oil) reaches 200–400 mg/person [84], in US the average daily intake of squalene is about 30 mg/person. Incidence of the breast cancer in Greece is 65% lower than in USA [85]. Analysing statistical health studies from Greece, Spain, and Italy comparative with USA, Newmark [86] suggested that this protective effect of olive oil consumption could be related to the high concentration of squalene in olive oil.

Another ancient plant came more recently into the attention of the researchers, for the same reasons:* Amaranthus* sp. It was widely used in nonconventional medicine by many people in the history. One of the first mentions of amaranth as a means of cleaning stomach and intestines can be found in the works of medieval Armenian doctor of the 16th century Amasiatsy [87]. Decoction of the leaves of* A. retroflexus* and* A. lividus* was recommended to cure headaches and tumors, mentioning the same reference.

Squalene is a natural triterpene known as an important intermediate of cholesterol/phytosterol biosynthesis in animal and plant organisms. Synthesis of squalene is similar in all organisms, although the enzymes implicated in its formation can have different properties. A short review of its biosynthesis will be helpful in understanding the squalene use in the prevention and treatment of various conditions.

In animal cells* de novo* cholesterol biosynthesis follows the mevalonate (MVA)/isoprenoid pathway. It starts with the conversion of acetyl-CoA to 3-hydroxy-3-methylglutaryl coenzyme A (HMG-CoA), followed by the reduction to MVA with 3-hydroxy-3-methylglutaryl coenzyme A reductase (HMGR). This is a rate limiting step, highly regulated through the HMGR activation or degradation [88]. The formation of isopentenyl diphosphate (IPP) and dimethylallyl diphosphate (DMAPP) occurs after phosphorylation and decarboxylation of mevalonate, DMAPP being a precursor of all polyprenyl compounds. The subsequent condensation with another IPP will conduct to farnesyl pyrophosphate (FPP). FPP can be further converted to squalene and sterols or involved in the synthesis of isoprenylated cellular metabolites [89].

In plants, the biosynthetic reactions from squalene to phytosterols lead to the formation of different sterols, like sitosterols, stigmasterol, and campesterol. First, squalene is oxidized to 2,3-oxidosqualene and then transformed to cycloartenol, the vegetable equivalent of lanosterol. Further, cycloartenol (9b, 19-cyclo-24-lanosten-3b-ol) is metabolized to the end product of this biosynthetic way, sitosterol [90].

The isoprenoids of plants can be synthesized either via MVA pathway, in the cytosol where sterols and brassinosteroids are formed and in mitochondria where side chains of ubiquinone are formed, or by 2-C-methyl-D-erythritol-4-phosphate (MEP) pathway, in plastids where carotenoids, plastoquinones, and isoprenoid phytohormones are synthesized [91]. By the MVA pathway only IPP is formed, while in the MEP pathway, IPP and also DMAPP are formed.

The MVA cycle and squalene biosynthesis in plants are quite similar to those met in animal metabolism, with three important steps catalyzed, respectively, by HMGR, farnesyl pyrophosphate synthase (FPS), and squalene synthase (SQS). The first step in MEP pathway is the condensation of glyceraldehyde-3-phosphate and pyruvate to form 1-deoxy-D-xylulose-5-phosphate (DXP). MEP is formed via reductive isomerization thereof catalyzed by 1-deoxy-D-xylulose-5-phosphate reductoisomerase (DXR). Further, MEP is transformed to IPP and DMAPP, condensation of two molecules of IPP with DMAPP, leading to FPP, step catalyzed by FPS. In the last step, two molecules of FPP condense and form squalene via presqualene diphosphate, step catalyzed by SQS [92].

Although squalene is an intermediate product in the cholesterol biosynthesis in humans, its daily consumption does not increase the cholesterol levels. The results of a clinical trial [93], conducted on elderly patients suffering from hypercholesterolemia, showed, by contrary, a decrease in total cholesterol, LDL cholesterol, and TAG levels and an increase of HDL cholesterol. Other reports [94] showed that an amaranth oil diet, also known to contain high concentrations of squalene, produces health benefits by decreasing or even disappearance of headaches, weakness, and increased fatigue. An issue still in dispute today is the fact that a daily diet containing amaranth oil reduces the serum cholesterol due to the action of squalene, important constituent of amaranth oil. Shin et al. [95] performed some experiments on rats with amaranth grain, oil, and squalene of different origins in order to study their hypocholesterolemic effect. They observed a different effect of squalene from amaranth source versus squalene from shark origin: the vegetal squalene proved a hypolipidemic action in blood and liver and an increase of cholesterol excretion in feces, effects that were not observed when shark squalene was administrated.

At the beginning of 1950s it was discovered that squalene is an important component of human sebum, a fact that justifies its role in the physiology of skin: its action in skin hydration, repairing of the damaged skin, and rejuvenating the ageing skin was demonstrated. The emollient and hydration properties of squalene and also its biocompatibility with skin make squalene an important component in cosmetical formulations (moisturizing creams, makeup, lipstick, and nail and hair products) [96]. It is considered one of the greatest natural emollients, being rapidly and efficiently absorbed into the skin, restoring its natural suppleness and flexibility, without back oily residues. All these characteristics make it an excellent skin protector, being used in healing eczema, damaged hair, and antiaging and wrinkle protection.

Furthermore squalene appears to play an essential role in protecting skin from free radical oxidative damage. Squalene acts in skin as a quencher of singlet oxygen, protecting by this mechanism the skin surface from lipid peroxidation due to exposure to UV light. Kohno et al. [97] showed in their experiments that the constant rate of quenching of singlet oxygen by squalene is much higher than those of other lipids in human skin and is comparable to that of 3,5-di-t-butyl-4-hydroxytoluene (BHT). They also stated that it seems to be unlikely to the chain reaction of lipid peroxidation in human skin to appear when proper levels of squalene are present.

Due to its antibacterial properties, squalene single or in admixture with squalane is used for preparing a cooling composition for the local treatment of burns [98]. Squalene has a melting point lower enough to allow the cooling composition to remain liquid, even at temperatures between −10°C and −60°C, unlike the ordinary oily topical drugs. The last mentioned ones are not preferred anymore as they become solid at these very low temperatures, or, at higher temperatures, they have not the desired cooling effect after burning. It was shown that topical application of a composition containing squalene, squalane, or mixtures thereof did not yield to irritations and had the desired effective action.

Squalene was also used as an adjuvant in vaccines, stimulating the immune response and increasing the patient's response to vaccine. It is added to lipid emulsions as drug carrier in vaccine applications [99]. The oil in water emulsions containing squalene facilitates solubilization and modifies the release and cell uptake of drugs, adjuvants, and vaccines. The experiments conducted by Kim et al. [100] on a mouse model demonstrated that a squalene emulsion had the most potent transfection activity and proved the least cytotoxicity after the intravenous administration. The most known today adjuvant including squalene is MF59 which belongs to Novartis, as patented compound. It comprises squalene together with two surfactants, Tween 80 and Span 85, as an oil-in-water microemulsion. It is used as adjuvant in several vaccines against hepatitis B and C, herpes simplex virus, and influenza virus. It was the first oil-in-water influenza vaccine commercially used [12]. Data published by the World Health Organization [101] showed that squalene was present in over 22 million flu vaccines distributed to patients in Europe since 1997 and no adverse effects were reported.

The already well-known antioxidant activity of squalene seems to be due to its chemical structure: a highly unsaturated isoprenoid hydrocarbon, containing 6 double bonds. Due to this double bond structure, this isoprenoid hydrocarbon acts as a strong antioxidant and a natural antibiotic. Also as a consequence of its biochemical structure, it is extremely reactive to get into the oxidized form. The unsaturated carbons of squalene bind hydrogen ions from water and release 3 unbound oxygen molecules, providing the saturated form squalane, C_30_H_62_. Due to this reaction, the oxygen reaches the cells, the cellular metabolism is intensified, the function of certain organs, like liver and kidney, is enhanced [102], and finally the vitality rises.

Squalene is not particularly susceptible to peroxidation and it is stable against peroxide radicals attacks that is why a protective effect of skin exposed to UV radiation is obtained when appropriate levels of squalene are present in the skin [103]. Its total concentration in the skin and the squalene to cholesterol ratio vary with the skin site [104].

The incidence of CHD is very high today due to various factors, including the modern life style. The studies performed in Mediterranean area suggested that a diet rich in olive oil reduces the incidence of CHD and cholesterol conditions, and squalene was supposed to be one of the factors responsible for these effects. Only a few reports are available regarding the effects of a diet containing squalene administrated to humans [93, 105]. According to these reports, following a supplementation of squalene of about 850–900 mg to the daily diet and taking into account that squalene is an intermediate in cholesterol biosynthesis in humans, no higher levels of cholesterol in serum were reported, although the concentration of squalene in serum rose up to about 17 times. The proposed explanation was the important growth of cholesterol elimination in feces which compensates the increased rate of cholesterol biosynthesis by squalene.

Experiments on animals suggested the protective effect of squalene against CHD, explained by its effect to inhibit the isoprenaline-induced lipid peroxidation [106].

The cancer chemotherapy has multiple undesired side effects, like the damages of the normal healthy tissues, even organ toxicity, toxic secondary effects which will finally limit the anticancer drug dosage, and even the treatment failure. Many of the anticancer therapies, chemical or by radiation, will produce free radicals, considered responsible for the mentioned toxic effects. Squalene already proved to be effective as an antioxidant. This was one important reason for which the natural antioxidant squalene to be experimentally tested; it proved to be a well-tolerated, nontoxic, and good cytoprotective agent [107].

The primary application of squalene in cancer therapy seems to be a potentiating agent for the anticancer drugs. There are reports about the good results obtained by testing squalene on animal models, in combination with antitumor agents as ACNU (3-[(4-amino-2-methyl-5-pyrimidinyl)methyl]-1-(2-chloroethyl)-1-nitrosourea) [108] or as bleomycin [109], against different cancer tumors. So far, no experimental trials on humans have been reported in order to confirm data obtained on animal models. Experimental data existing up to now could indicate that squalene is involved in the biochemical way by which anticancer drugs act. It seems that squalene may stop the tumor cells' development or prevent some forms of chemically induced cancer and even produce regression of existing tumors in some cases. The suggested mechanism by which squalene could inhibit tumor formation implies either its inhibitory effect on the catalytic activity of *β*-hydroxy-*β*-methylglutaryl-CoA (HMG CoA) reductase and subsequent inhibition of farnesylation of Ras oncoproteins or modulation of the biosynthesis and of the functional activity of the enzymes involved in xenobiotic metabolism or its action as a free radical scavenger [1].

## 5. Conclusions

Squalene is considered today an interesting natural molecule, with broad applications in food industry and cosmetics and in prevention and treatment of human diseases. In order to preserve the marine life, new accessible natural sources of squalene and cost-effective methods to separate it are sought. Reviewing the existing literature data, we concluded that deodorizer distillates from the distillation of edible vegetable oils like olive, soybean, or palmer oils represent a valuable natural source for squalene. Amaranth oil could be considered an interesting source as the squalene concentration in this oil is probably the greatest in the vegetable kingdom, but the process is cost-effective only if it is coupled with the obtaining of starch rich flour from* Amaranthus* seeds or leaves. In terms of separation methods, the conventional method of solvent extraction still remains the most preferred in the industry for the oil extraction, followed by molecular distillation for the squalene separation. Supercritical fluid extraction with CO_2_ represents an environmentally friendly process which is now used only at laboratory or pilot level, especially for the oil fractionation in order to separate valuable minor components, like squalene or tocopherols. The analytical techniques allow today the quantitative determination of squalene in the presence of other components present in the deodorizer distillate.

## Figures and Tables

**Table 1:** Chemical and physical properties of squalene.

Properties	Value
Molecular weight	410.7 g mol^−1^
Melting point	−75°C
Refractive index	1.499
Viscosity at 25°C	12 cP
Density	0.858 g/mL
Boiling point at 25°C	285°C
Flash point	110°C
Iodine number	381 g/100 g
Infrared peaks	2728, 1668, 1446, 1380, 1150, 1180, 964, 835 cm^−1^
Surface tension	~32 mN/m

**Table 2:** Chemical composition of some crude vegetable oils [28, 30–33].

Oil	FFAs (% wt)					Total sterols (mg/100 g)	Tocotrienols + tocopherols (mg/100 g)	Squalene (mg/100 g)	Unsaponifiable (% wt)
	16:0	18:0	18:1	18:2	18:3				
Soybean	1	2	78	7	1	182	200		0.6
Olive	44	4	39	11	Traces	65	900	150–170	0.7
Palm	11	4	22	53	8	318	1,370	25–54	0.1–0.34
Amaranth	22	3	29	45	2		2.8–7.8	6,000–8,000	5.93

**Table 3:** Chemical composition of most common edible oil deodorizer distillates (% wt).

Type	FFA	Sterols	Hydrocarbons	Other substances	Reference
Soybean	30–60	10–35	10–30	7	[39]
Olive	34.2	4.6	31.5 (SQ-28%)	5.6	[40]
Palm	20.34	4.77	3.94—as SQ	62.12	[28]

**Table 4:** Comparative separation methods of squalene from amaranth oil.

Separation method	Concentration g/100 g
SFE	6.95
Cold pressing	5.74
Extraction with chloroform/methanol	6.00

**Table 5:** Methods for squalene separation from natural sources.

Natural source of squalene	Procedure/conditions	Observation	Reference
Olive oil	Process involving molecular distillation during industrial deodorization in refining process of olive oil	Recovery of all minor components	[51]

Deodorizer distillate (DD)-vegetable oils	(i) DD dissolved in different solvents (3 : 1, w/v), like methanol and acetone (ii) Crystallization at −20°C, 24 h, mixture of acetone and methanol (4 : 1) (iii) Centrifugation, filtration, washing solvent: DD = 3 : 1 (v/v)	93% recovery of squalene from DD (2 washes) in filtrate; 79.5% recovery of total sterols in the cake	[52]

Olive biomass	Pressurized fluid extraction (PPE) (Soxhlet) (i) Acetone, 100°C, 3 × 10 min (ii) 2-propanol, 190°C, 3 × 10 min	(i) 575 *μ*g squalene/g (ii) 509 *μ*g squalene/g	[53]

*Amaranthus* grain	(i) Extraction of oil from flour in a Soxtec System HT6 with petroleum ether (40–60°C) (ii) Remove the unsaponifiables with a mixture of EtOH 95% : KOH 50% (30 : 5; v/v) (iii) Unsaponifiables extraction 5x petroleum ether; washing, drying, filtration, solvent removing (iv) Isolation of squalene from ultrafiltrate by column chromatography on a silica gel (24 g, 70–230 mesh, Sigma Co.) column	=>squalene content increased from 4.2% in crude oil to 43.3% in the unsaponifiables (i) 94% purity squalene (ii) 90% recovery	[54]

Vegetable oil (palm oil)	Extraction and isolation of minor components following the steps (a) Esterification of the oil with an alcohol (b) Collection of the esters phase from glycerol (c) Distillation of the ester phase (d) Dilution of the concentrate in a nonpolar solvent (*p* = 0.2–50 barr) (e) Adsorption of the concentrate on an adsorbent, (f) Extraction of the minor components using a mixture of solvents (g) Desorption of the minor components by a mixture of solvents	(a) MeOH (c) Short path distillation at *T* < 200°C, *p* < 150 mmTorr => concentrate with 5% carotene, 0.5% tocols, 0.7% squalene, 0.7% sterols (d) Hexane (e) Normal-phase silica gel packed column (12 cm × 4 cm i.d.); feed/adsorbent = 23 g/kg (f) Hexane : isopropanol = 99.5 : 0.5, *p* < 1 barr (g) First fraction collected is squalene Squalene recovery = 92%	[41]

Olive oil (residues)	Process comprising four steps: saponification, cracking and esterification of the fatty acids, and extraction by super and/or subcritical gases into a high pressure extraction column with independently temperable column regions	Squalene with >90% purity	[55]

Animal source, like shark liver oil or an extract thereof	Method comprising steps (a) Purification distillation at *T*_1_ (b) Denaturing distillation at *T*_2_, *T*_1_ and *T*_2_ are temperature to cause squalene boiling; *T*_1_ < 140°C and *T*_2_ ≥ 200°C (c) saponification of the composition (shark liver oil or extract thereof) prior to or after distillation stages	(a) *Tϵ* [70°C, 100°C] *pϵ* [0.5 *μ*mHg, 5 *μ*mHg] composition with >95% squalene (b) *Tϵ* [200°C, 300°C] *pϵ* [0.5 mmHg, 5 mmHg] composition with at least 99.9% squalene	[56]

Deodorizer distillates from plant oils	Process for the simultaneous extraction of squalene, sterols, vitamin E (1) Esterification of FFA (2) Transesterification of the combined FA with the same short alcohol (3) Three successive distillations; third distillate contains squalene	(1) Alcohol C_1_–C_3_: fatty acids in a molar ratio >5 Acid catalyst : distillate = 1 : 10 (w/w) *T* < 95°C (2) Alcohol C_1_–C_3_, basic catalysis, *T* < 100°C (3) First distillation on a column with 20 theoretical plates, *p* = 3 mbarr–10 mbarr; *T* = 160°–180°C Second distillation on a column with 10 theoretical plates, *p* = 20 mbarr–30 mbarr, *T* = 220–225°C Third distillation on a column with 10 theoretical plates; *p* = 1 mbarr–10 mbarr, *T* = 220–260°C	[57]

*Amaranthus *grain	(i) Supercritical CO_2_ extraction with or without cosolvent EtOH (2% or 5%) (ii) Extract fractionation by gradual decrease of pressure yields (at ambient conditions) to the highest concentration of squalene	(i) At 55 MPa and 5% cosolvent: 0.289 g squalene/100 g seeds (ii) Up to 17.9 g SQ/100 g oil (iii) Tocopherols were also extracted in the same process	[50]

Crude palm oil (CPO)	Extraction of phytosterols, squalene, vitamin E, following the steps (a) Conversion of crude palm oil in palm oil methyl esters (b) 3 short path distillations of PO methyl esters (c) Saponification of phytonutrients obtained in stage (b) (d) Crystallization of phytosterols (e) Solvent partitioning of vitamin E and squalene	(a) MeOH/EtOH with NaOH/KOH; (b) I short path distillation at *T*: 70°C–120°C and *p*: 1.33 Pa–6.67 Pa II short path distillation at *T*: 130°C–200°C and *p* < 1.133 Pa III short path distillation at *T* < 120°C and *p* < 1.133 Pa (c) 10% NaOH/KOH (d) unsaponifiable fraction obtained in step (c) mixed with hydrocarbon solvent/C_1_–C_4_ alcohol/H_2_O = 25/1/1/, heat at 65–85°C, cool slow at 25–30°C (e) mixing filtrate from (d) with heptane/hexane/i-octane and C_1_–C_4_ alcohol (5 : 3); squalene extraction from hydrocarbon layer and vitamin E extraction from alcohol layer; original CPO contains 250–730 ppm squalene, recovery by this process up to 97%	[58]

Residues from olive oil deodorization process	Countercurrent supercritical CO_2_ extraction	Isothermal countercurrent column, without reflux, at *T* = 343 K, *p*: 150–230 bar, solvent : feed ratio = 13 : 1; A product with up to 90% (wt) squalene was obtained	[59]

OODD (olive oil deodorization distillate)	Supercritical fluid extraction	(1) Esterification in supercritical MeOH (2) Extraction of squalene with SC-CO_2_ at 52.05°C, *p* = 104.8 bar, extraction time 180 min A raffinate with 75% squalene was obtained	[35]

OODD	Supercritical CO_2_ extraction	(i) Saponification of FFAs with glycerol and acid catalyst (Zn) (ii) Glycerol esterified product is subject to SC-CO_2_ extraction: *T* = 40°C, *p* = 150 bar, 30 Kg CO_2_/Kg sample, countercurrent column (3 m × 30 mm, i.d.) with Sulzer stainless steel rings as packing material; extract with 83.7% squalene; extraction yield = 83.7% (iii) Column with temperature gradient SC-CO_2_extraction 50 : 40 : 30 → 90% squalene in extract; extraction yield = 91.1% (iv) Pilot plant scale experiments	[40]

Palm fatty acid distillate	Supercritical fluid extraction	(i) 50 mL extractor (320 mm × 13 mm, i.d.) with glass beads packing; (ii) SC-CO_2_ extraction at 3 mL/min for 90 min, at 200 bar and 50°C (iii) 438.16 ppm squalene	[60]

SODD (soybean oil deodorization distillate)	Supercritical CO_2_ distillation-extraction	(i) *T*: 50–90°C; *p*: 24.1–31.0 MPa; 200–1000 L STP CO_2_; (ii) Concentration factor for squalene in extract = 1.26	[61]

Amaranth oil	Short path distillation	(i) Short path distillation unit (KDL4 Model, UIC Inc., Joliet, IL) (ii) degumming of crude oil by heating at 90°C, adding 2% (w/w) H_2_O, stirring, resting for 24 h, centrifugation at 18,900 g for 20 min; (iii) Alkali-refining of the degummed oil with 20% (w/w) caustic soda (iv) Best result for fractionation by short path distillation at *T* = 180°C and 3 mtorr: (i) squalene concentration in distillate increased sevenfold, with 76% recovery	[44]

Mutant yeast obtained by genetic engineering techniques	Supercritical CO_2_ solvent extraction	(i) Two-stage cultivation system for yeast: aerobic for biomass growth, anaerobic for squalene production (ii) Disruption of cells in a glass bed mill; (iii) Separation of squalene in two steps (a) Solvent extraction from lysate with CHCl_3_ : MeOH = 2 : 1 (v/v) (b) Lyophilization, followed by SC-CO_2_ extraction → 95% purity squalene	[62]

*Torulaspora delbrueckii *biomass	Supercritical CO_2_ extraction	(i) Cells after anaerobic fermentation subject to lyophilization; (ii) SFE at *T* = 60°C, *p* = 250–255 bar, CO_2_ flow rate: 0.2 L/min (iii) Yield: 430.52 *µ*g squalene/g dry weight cells	[63]

EOODD (by-product obtained after distillation, esterification and transesterification of OODD)	Countercurrent SFE (supercritical fluid extraction)	(i) *T* = 343 K, *p* = 180 bar, solvent (CO_2_)/feed ratio = 13 → 84% (wt) purity squalene, 64.2% yield; (ii) Countercurrent extraction column with 15 theoretical plates, with reflux, *T* = 351 K, *p* = 177 bar, solvent/feed ratio = 51, reflux/extract ratio = 3.6 → 92% purity squalene, 93% yield	[64]

*Terminalia catappa *leaves and seeds	Supercritical CO_2_ extraction	(i) Preliminary treatment for leaves: cutting, freeze-drying, grinding (ii) Extraction at *T* = 40°C, *p* = 3,000 psi, static extraction for 15 min, flow 3 mL/min → high purity squalene	[65]

**Table 6:** Summary of the analytic techniques used in quantification of squalene in the presence of acylglycerols, FASEs, free phytosterols, and tocopherols, from natural sources.

Technique	Conditions/observation	Reference
Densitometric estimation	Solvent: cyclohexane (Rf value SQ = 0.60 ± 0.02); SQ spots detected with iodine vapours; determination on a HPTLC unit at *λ* = 200 nm	[72]

GC	Capillary column CP-Sil 8 CB (15 m, 0.1 mm, i.d. 0.25 mm, Chrompack, Middleburg, The Netherlands), oven temperature program = 60–140°C at 30°C/min and 340°C at 15°C (15 min hold); carrier gas = Helium at 41.3 kPa; FID temperature = 360°C	[73]

HT-GC	TLC + GC-FID (Shimadzu 17A, Japan); separation on a DB-5HT (5%-phenyl)-methylpolysiloxane nonpolar column (30 m, i.d. 0.32, Agilent Tech. Palo Alto, US); temperature program: injector and detector temperatures set at 370°C, column regime: 80–365°C at 15°C/min (8 min hold); split ratio = 1 : 50, using N_2_ as carrier gas, linear velocity = 30.6 cm/s at 80°C temperature = 200°C, range of *m*/*z* 30–600 at 1250 scan/s	[38]

GC	3800-GC (Varian, USA) with FID, DB 225 column (30 m, i.d. 25 mm), carrier gas N_2_, linear velocity = 34.8 cm/s, split ratio: 75, temperature programme: 180°C (1 min hold) to 220°C, with 3°C/min (2 min hold); detector temperature set at 300°C, injector temperature 290°C	[74]

HPLC	Hewlett-Packard 1100 HPLC system, RP-C_18_ column (Nucleosil 100-C_18_, 5 *µ*m, 250 × 4 mm i.d.), with photodiode array detector (*λ* = 214 nm for squalene); isocratic elution with methanol-isopropanol-acetic acid (91.95 : 8 : 0.05, v/v), flow rate 1.0 mL/min, RT for squalene 9.9 min	[54]

NIR	Visible/near-infrared scanning spectrophotometer (NIR Systems 6500, Perstorp Analytical Inc.); spectra recorded between 400 and 2,500 nm, at 2 nm intervals as (log⁡1/R), R = reflected energy	ibid

GC	Hewlett-Packard 3500 GC with FID, CP-TAP column (25 m × 25 mm i.d.,Varian, USA), split ratio = 1 : 50, carrier gas He, 1 mL/min; temperature programme: oven initial temperature 80°C (3 min hold), rising to 150°C at 10°C/min, to 250°C at 5°C/min, to 340°C at 10°C/min (20 min hold)	[75]

FT-IR	Perkin-Elmer-783 spectrophotometer, using CHCl_3_ as solvent; IR (neat, cm): 2914, 2728, 1668, 1446, 1382, 1330, 1224, 1151, 1188, 964, 835, 722.1	[72]

GLC	439 Packard model GLC with FID connected to a Chrompac CR-3A integrator; 2 m × 2 mm i.d., glass column packed with 10% SE30 on Chromosorb W; temperature program: 200°C (3 min hold), raised to 270°C at 5°C, detector and injector temperatures set at 320°C	[76]

HPLC	HPLC system (Waters Corporation, Milford, US), simultaneous determination of SQ and tocopherol, on Supelcosil LC-18-DB column (250 × 4.6 mm i.d.; Supelco, Bellefonte, US); mobile phase: 99% MeOH + 1% H_2_O, flow rate 1.2 mL/min; column temperature maintained at 25°C; chromatograms extracted at 292 nm tocopherol, 215 nm SQ	[77]

RPLC-GC	Hewlett-Packard model 1050 LC (Wilmington, DE) with manual injection valve (model 7125, Rheodyne, Cotati, CA) having a 20 *µ*L loop, coupled with a Perkin-Elmer model 8500 GC (Norwalk, CT) with PTV injector (Perkin-Elmer) and FID. LC conditions: mobile phase = methanol/water, 50 × 4.6 mm i.d. column slurry packed with 10 *µ*m silica, C_4_, Vydac 214 TPB, UV detection at 205 nm, LC column maintained at 45°C; mobile phase regime: methanol/water = 70 : 30 (hold 3 min) linear decrease to 22% water in 3 min, maintained 4 min, linear decrease in 2 min to 14% water, kept 3.5 min, to 0% in 3.5 min. GC conditions: 5% diphenyl/95% dimethyl polysiloxane fused silica column (30 m × 0.25 mm i.d., Sugelabor, Madrid, Spain), carrier gas = He; temperature programme: 130°C–230°C at 20°C/min, maintain 2 min, raise at 290°C at 3°C/min, maintain 30 min, detector temperature set at 320°C	[78]

Colorimetric method	Rapid method for quantitative determination of 10–150 *µ*g squalene: (i) solution containing squalene is reduced to dryness under nitrogen, add H_2_SO_4_, maintain in water bath at 70°C, for 5 min → pale yellow colour develops, adding formaldehyde to intensify and stabilize the colour; (ii) optical density determination at *λ* = 400 nm	[79]

Mass spectrometry	HPLC coupled to electrospray tandem mass spectrometry, rapid and selective method for SQ determination in olive oil	[80]

EA-IRMS	Elemental analyser coupled to isotope-ratio mass spectrometer for detection of SQ origin (vegetal/animal); Thermo Scientific Flash 1112 EA for IRMS coupled to a Thermo Scientific Delta V Series IRMS via a Thermo Scientific ConFlo IV interface; duration for squalene/squalane analysis is 400 s	[81]
